# Consequences of multiple simultaneous opportunities to exploit others’ efforts on free riding

**DOI:** 10.1002/ece3.6201

**Published:** 2020-04-16

**Authors:** Frédérique Dubois, Étienne Richard‐Dionne

**Affiliations:** ^1^ Department of Biological Sciences University of Montreal Montreal Quebec Canada

**Keywords:** evolutionary stable strategy, game theory, producer–scrounger game, simultaneous scrounging opportunities, stochastic effects

## Abstract

Individuals within a group do not all act in the same way: Typically, the investors (or producers) put efforts into producing resources while the free riders (or scroungers) benefit from these resources without contributing. In behavioral ecology, the prevalence of free riders can be predicted by a well‐known game‐theoretical model—the producer–scrounger (PS) model—where group members have the options to either search for resources (producers) or exploit the efforts of others (scroungers). The PS model has received some empirical support, but its predictions, surprisingly, are based on the strict assumption that only one resource can be exploited at a time. Yet, multiple simultaneous opportunities to exploit others’ efforts should frequently occur in nature. Here, we combine analytic and simulation approaches to explore the effect of multiple simultaneous scrounging opportunities on tactic use. Our analyses demonstrate that scrounging rates should increase with the number of simultaneous opportunities. As such, the amount and spatial distribution (i.e., clumped vs. dispersed) of resources as well as the risk of predation are key predictors of scrounging behavior. Because scroungers contribute to reducing the speed of resource exploitation, the model proposed here has direct relevance to the exploitation and sustainability of renewable resources.

## Graphical abstract

Free riding (i.e., the exploitation of others’ efforts) has a negative impact on the productivity of the groups but tends to reduce the speed of resource exploitation. Predicting the prevalence of free riders, therefore, may be important for the sustainability of renewable resources. Free riding behaviour has been investigated by the producer–scrounger (PS) game, where group members have the options to either search for resources (producers) or exploit others’ efforts (scroungers). Here, we developed a generalization of the PS model to multiple simultaneous discoveries.

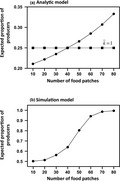

## 1 INTRODUCTION

Individuals, within groups (or populations), do not all behave in the same way and can therefore have different impacts on the productivity of their groups or on the sustainability of the resources they exploit. Typically, some individuals, referred to as investors or producers, put efforts into generating services or resources while others, referred to as free riders, freeloaders, exploiters, or scroungers, benefit from these services or resources without any contribution (Giraldeau, Heeb, & Kosfeld, 2017). Because the presence of such exploiters within a group is generally thought to reduce its performance, several modeling attempts have been made to identify the factors that affect free riding and understand their impact on individuals’ decision to generate resources or exploit others’ investment (Archetti & Scheuring, 2012; Dawes, 1980; Doebeli & Hauert, 2005). The producer–scrounger (PS) game, notably, has been frequently used by behavioral ecologists to predict the occurrence of free riding under different scenarios (Barta, 2017; Giraldeau & Caraco, 2000; Valone et al., 2017; Vickery, Giraldeau, Templeton, Kramer, & Chapman, 1991). In this classical example of the game‐theoretical approach, group members compete by scramble competition for access to resources and have the options, at any given time, to either search for resources themselves (producers) or exploit the efforts of others (scroungers). The original PS model assumes that a producer that discovers a resource gets a finder's advantage and then shares the remainder resource with all the scroungers. As such, the payoffs to scrounger are negatively frequency‐dependent: when scroungers are common, they do worse than producers, but when scroungers are rare, they do better than producers. Therefore, the solution of the game is a mixed evolutionary stable strategy (ESS) in which producers coexist with scroungers and both tactics obtain the same payoff.

The PS game model has been extremely successful in predicting changes in PS tactic use in response to changes in several ecological and social factors (see Afshar & Giraldeau, 2014; Giraldeau & Dubois, 2008). In particular, it predicts an increase in producer use with an increase in the finder's share (i.e., the proportion of food consumed by the producer to the total amount of food in the patch). Accordingly, several studies have demonstrated that the proportion of producers in a group is higher when the food is scattered into a large number of poor food patches, and the producers therefore consume a large fraction of the food before the scroungers arrive, rather than clumped into a few rich patches (Coolen, Giraldeau, & Lavoie, 2001; Hansen, Ward, Furtbauer, & Kink, 2016; Morand‐Ferron & Giraldeau, 2010; Morand‐Ferron, Varennes, & Giraldeau, 2011). Yet its predictions, surprisingly, are based on the strict assumption that only one resource can be exploited at a time (Vickery et al., 1991; Giraldeau & Caraco, 2000, Barta & Giraldeau, 2000; but see Ohtsuka & Toquenaga, 2009) and, as such, are unaffected by the rate of resource discovery. Such an assumption is correct, for example, when the probability of finding food is so small that patch discoveries are extremely rare events, when the resource discovered by a producer is exploited instantaneously or when the producers all contribute to the same good. It is most likely, however, that new food patches are frequently discovered while others have not yet been fully exploited, thus providing scroungers with the opportunity to disperse over several patches. The occurrence of multiple simultaneous scrounging opportunities should then dynamically change the costs and benefits of producing, and consequently, affect the benefits and frequency of scrounging.

Particularly, if different resources are simultaneously available and scroungers decide randomly which resource to exploit, the fraction of each new resource that is consumed by these individuals will decrease as the number of simultaneous discoveries increases, leading to an increase in the payoff to producing. Conversely, the occurrence of multiple simultaneous scrounging opportunities should negatively impact the scroungers’ payoff, by reducing the number of patches they can join per time unit. Every factor that affects the rate of resource discoveries should then directly affect the amount of scrounging in a group, with less scrounging that should be expected when the conditions favor more simultaneous opportunities to scrounge. Because experimental tests of the PS game always focus on one subject at a time, thereby ignoring what the other group members simultaneously do, there are no available data on the frequency of multiple scrounging opportunities and so no direct evidence yet that such events may profoundly impact scrounging rates. However, results from several experiments (Barrette & Giraldeau, 2006) and simulation models (Afshar & Giraldeau, 2014; Beauchamp, 2008; Beauchamp & Giraldeau, 1996) found that scrounger tactic use tends to decrease as the encounter rate with food patches, and likely the number of simultaneous scrounging opportunities, increases (Afshar & Giraldeau, 2014). Furthermore, as anticipated, a variant of the PS model used to predict the change from solitary to social foraging and that considered multiple simultaneous discoveries reported that the proportion of producers increased with the encounter rate with food patches (Ohtsuka & Toquenaga, 2009). This model, however, has no analytic solution and may produce different predictions for a given set of parameter values. Furthermore, the rate of resource discovery is fixed in Ohtsuka and Toquenaga’s (2009) analysis and as such it cannot explicitly explore the effects of predation pressure or interference competition on tactic use, though these factors can directly influence the time spent searching for food. Specifically, a lower prey‐discovery rate at higher densities is frequently observed as increasing the number of competitors in a group tends to increase the time individuals spent interacting but decrease the time they spent searching for food (Ens & Goss‐Custard, 1984). Both the strength and the direction of the association between group size and prey‐discovery rate, however, can vary depending on the spatial distribution of the prey or the perceived risk of predation. In environments with high predation risk, particularly, increasing group size can lead to a decrease in the time spent scanning, thereby allowing individuals to increase their efficiency (Pulliam, 1973). Depending on ecological conditions, therefore, the number of multiple scrounging opportunities could then potentially increase or decrease with increasing group size.

To further explore the effect of multiple scrounging opportunities on free riding behavior, here, we then set out to develop a modified version of the PS model in which the best use of the producer and scrounger tactics depends on the average number of simultaneous scrounging opportunities. Because the model is based on simplifying assumptions to be tractable, however, we also use a stochastic simulation model to confirm the qualitative predictions from the analytic model. As anticipated, the analytic model predicts that the equilibrium proportion of scroungers should decrease as the number of simultaneous scrounging opportunities increases. For this reason, both approaches predict that the frequency of scrounging should be affected by the amount and distribution of resources as well as the time devoted to searching for food, which is determined notably by the risk of predation. In addition, while the original PS game predicts more scrounging in larger groups, our analyses demonstrate that increasing group size can either mitigate or exacerbate scrounger tactic use if the rate of resource discoveries increases or declines as the proportion of producers increases, respectively. Finally, although the analytic model still converges toward an equilibrium value, the mean payoffs to producing and scrounging are expected to vary over time depending on the number of resources available. As a consequence, we might expect fluctuations in mean tactic use because of stochastic effects.

## 2 A PS GAME WITH SIMULTANEOUS SCROUNGING OPPORTUNITIES

For simplicity, the game is set in an explicit social foraging context but it can be generalized to other systems of exploitation. Following Giraldeau and Caraco (2000), we consider a foraging group in which each of the G members can search either for food (thereby using the producer tactic) or for opportunities to exploit others’ discoveries (thereby using the scrounging tactic). The proportion of producers and scroungers is denoted by *p* and (1 − *p*), with 0 ≤ *p *≤ 1. For simplicity, we assume that the time required to exploit a patch is negligible. Thus, we consider that all *n* producers (with n=p×G) actively search for food at any time and that the scroungers distribute themselves among all available patches. This simplifying assumption was made to keep the model tractable, but may generate estimates of the average number of simultaneous scrounging opportunities that are upward biased. This is because producers that do exploit a patch that is not depleted yet do not actively search for food. However, as previously discovered, patches still represent opportunities to scrounge until they are not depleted, and all producers can then generate one scrounging opportunity at any time even if they discovered a food patch in a previous time step, the qualitative predictions from the analytic model are robust to these errors. Accordingly, they were all confirmed by a stochastic version of the analytic version of the model (see next section), in which the producers could at any time either search actively for food or exploit a previously discovered food patch.

At any time, all individuals using the producer tactic have a probability *f* of discovering a food patch and a probability (1‐*f*) of not finding a patch, with:

(1) $$f=\frac{N_{F}}{N_{\text{SA}}}\times \left[\frac{1}{1+\exp(\alpha n)}\right]$$

In this equation, *N_F_* and *N*_SA_ (with 0 < *N_F_* < *N*_SA_) represent the number of food patches and the total number of potential patches (i.e., the size of the search area), respectively, while *α* is an interference parameter. Because the model assumes that the resources are renewed at the same rate as they are exploited, the probability of discovering a food patch (*f*) remains constant over time, for a fixed number of producers. The rate of resource discovery, however, decreases or increases as the number of producers increases when α is greater or smaller than zero, respectively.

As discovered food patches are replaced, the probability that *k* patches are simultaneously discovered follows a Binomial distribution and the average number of simultaneous discoveries $\bar{k}$ can be estimated as:

(2) $$\bar{k}=n\times f$$

Food patches all contain *F* food items. When a producer finds a patch, it gets *a* items (i.e., the finder's advantage) before the scroungers arrive, and then, the remaining (*F* − *a*) food items are shared between the producer and all the scroungers who joined the patch.

Knowing, $\bar{k}$, the mean number of discoveries that are simultaneously available at any given time (with $\bar{k}$ > 0 given that *N_F_* > 0), we can estimate the average payoff to producers (*W_P_*) as:

(3) $$W_{P}=\frac{\bar{k}}{\mathrm{pG}}\times \left[a+\frac{F-a}{1+\left[\left(1-p\right)\times G\right]/\bar{k}}\right]$$

The first term of Equation (3) corresponds to the average probability that a producer discovers a food patch, assuming that the *p*G producers are all equally efficient at finding food, while the second term represents the average gain expected by a producer when it has discovered a food patch and has to share the remaining (*a* − F) items with, on average, $\left[\left(1-p\right)\times G\right]/\bar{k}$ scroungers.

Similarly, we can estimate the average payoff to scroungers (*W_S_*) as:

(4) $$W_{S}=\frac{F-a}{1+\left[\left(1-p\right)\times G\right]/\bar{k}}$$

Because it is assumed that the scroungers are capable of detecting every finding opportunity and randomly join one patch when several patches are simultaneously discovered, all scroungers, contrary to the producers, necessarily obtain a share of one discovered patch.

To estimate the equilibrium proportion of producers p*, we find the value of *p* for which both tactics have the same payoffs. Resolving *W_P_* = *W_S_* gives:

(5) $$p^{*}=\frac{a}{F}+\frac{\bar{k}}{G}$$

According to this equation, the proportion of producers at equilibrium is equal to: $p^{*}=\frac{a}{F}+\frac{1}{G}$ when $\bar{k}=1$, which corresponds exactly to the value predicted by the PS models that consider that only one resource can be discovered and exploited at a time (Giraldeau & Caraco, 2000; Vickery et al., 1991). As such, the present model is a generalization of the original PS model. In our model, however, the equilibrium value p*, given by Equation 5, depends on the average number of simultaneous discoveries ($\bar{k}$), which is determined by the proportion of producers in the group (*p*).

To find the solution of the game, we then use the best response dynamics. We consider an initial proportion of producers (*p*_0_), and we calculate the average number of simultaneous discoveries ${\bar{k}}_{0}$ using Equation (2). Knowing the average number of simultaneous discoveries, we then seek the optimal proportion of producers $p_{1}^{*}$, from Equation 5, and we repeat the same procedure until finding the solutions p* and $\bar{k}$* that satisfy the following conditions: $p_{t+1}^{*}=p_{t}^{*}=p^{*}$ and ${\bar{k}}_{t+1}^{*}={\bar{k}}_{t}^{*}={\bar{k}}^{*}$.

## 3 PREDICTIONS

**Figure 1:**
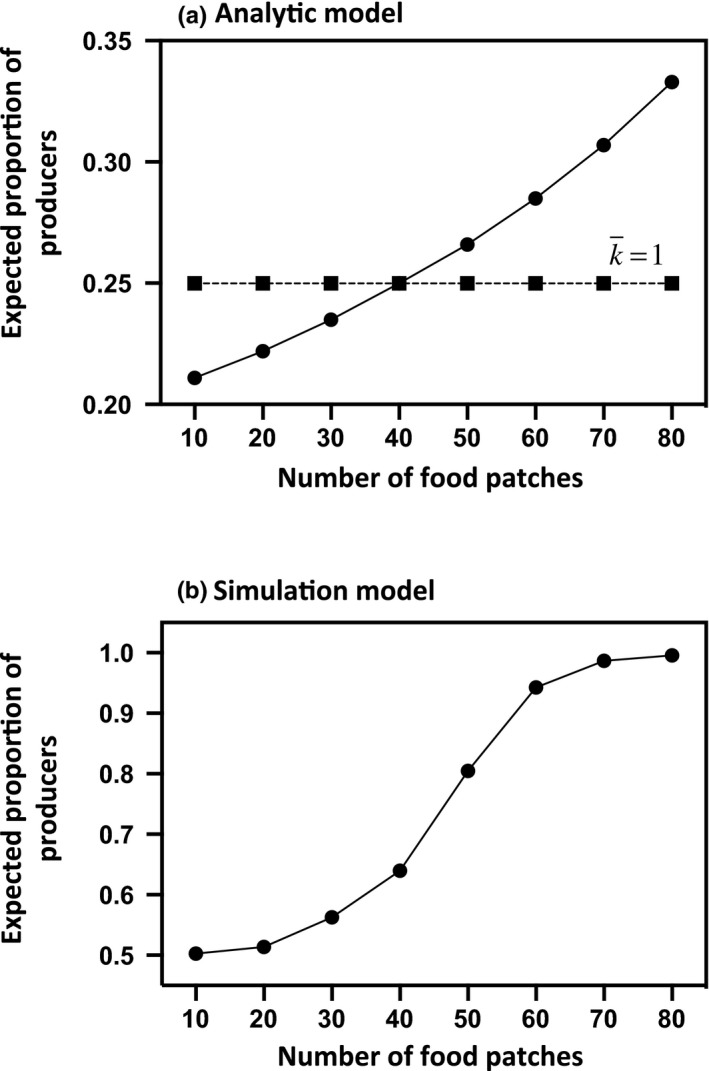
Expected proportion of producers at equilibrium in relation to the number of food patches that directly determines their probability of finding a food patch. Panel a: The number of simultaneous discoveries is fixed at 1 in Equation 5 (squares) or is calculated using the *technique of iterative best response dynamics* (dots). *F* = 10, *a* = 2, *N*_SA_ = 100, α = 0, G = 20. Panel b: *F* = 20, *N*_SA_ = 200, *G* = 20, *x* = 3, *δ* = 5

**Figure 2:**
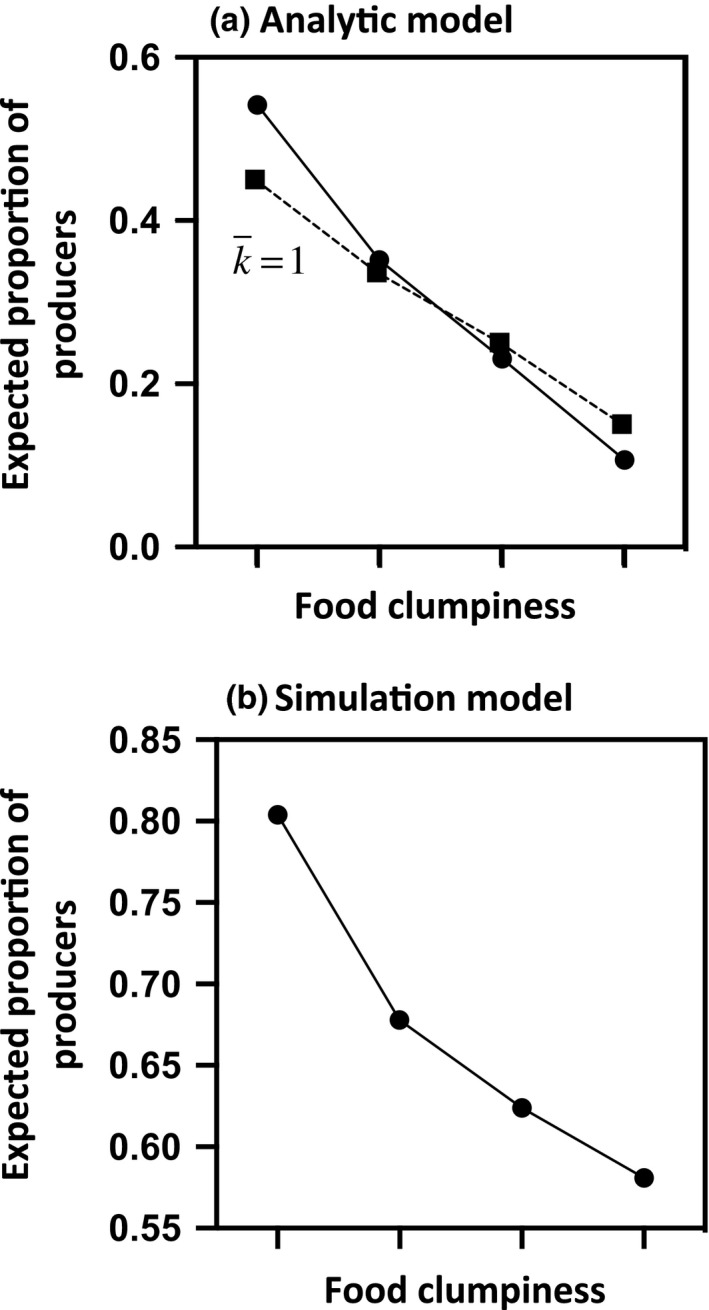
Expected proportion of producers at equilibrium in relation to the degree of clumpiness of the food. The total number of food items is always between 520 and 530, and the number of food patches is (from the left to the right) 105 (that each contains 5 items), 75 (that each contains 7 items), 53 (that each contains 10 items), and 26 (that each contains 20 items). Panel a: The number of simultaneous discoveries is fixed at 1 in Equation 5 (squares) or is calculated using the *technique of iterative best response dynamics* (dots). *a* = 2, *N*_SA_ = 200, α = 0, G = 20. Panel b: *N*_SA_ = 400, G = 20, *x* = 3, *δ* = 5

**Figure 3:**
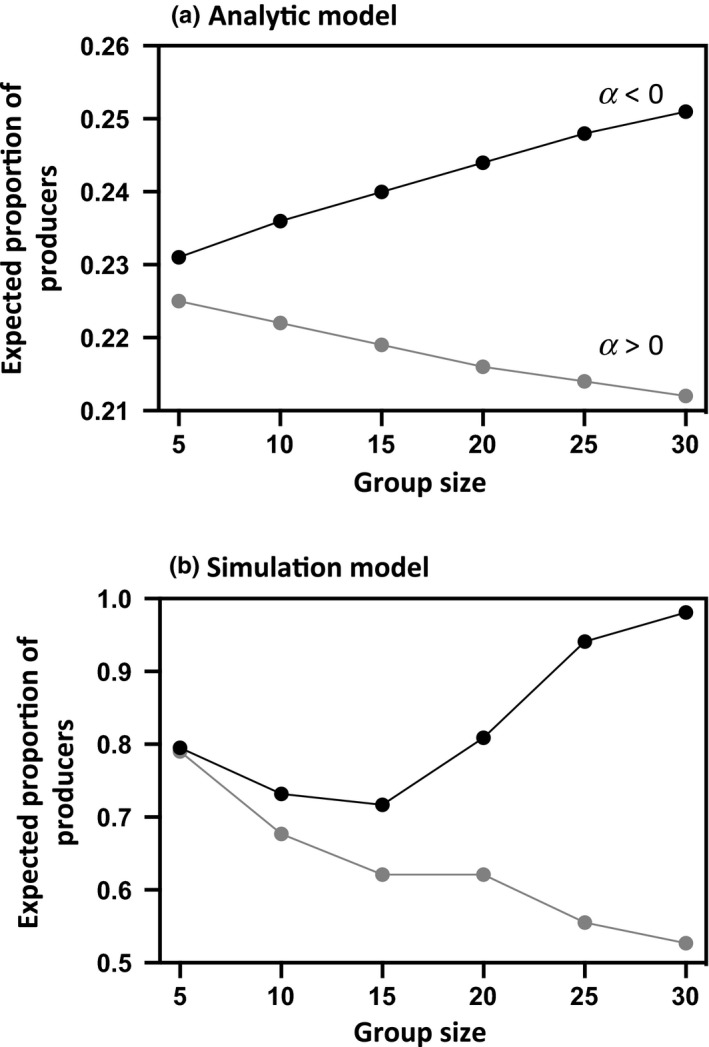
Expected proportion of producers at equilibrium in relation to group size. Panel a: The rate of discoveries decreases (*α* = 0.2, gray dots) or increases (*α* = −0.2, black dots) as the number of producers searching for food increases. *F* = 10, *a* = 2, *N_F_* = 50, *N*_SA_ = 200. Panel b: The number of patches a producer can assess during a time step (that directly determines its probability of finding a food patch) is fixed at 5 (gray dots) or increases with competitor number (*δ* = G, black dots). *N_F_* = 20, *N*_SA_ = 400, *F* = 20, *x* = 5

The model predicts that the group should always converge toward an equilibrium value that corresponds to the best response strategy for a fixed number of simultaneous discoveries. Yet, our analysis demonstrates that ignoring the number of discoveries that occur concurrently may lead the model to either overestimate or underestimate scrounging. More precisely, when the probability of finding a food patch is low, there is, on average, less than 1 food patch that is available at any time, giving rise to a smaller proportion of producers at equilibrium (Figure 1a). Inversely, when food patches are abundant and easy to find, one would expect individuals to invest more in the producer tactic (and then to scrounge less). The rate of resource discovery becomes then a key factor for predicting the amount of scrounging. Obviously, the probability of finding food depends on the quantity of resources available, but also on their spatial distribution and on the search area of the producers. Specifically, the model predicts that, for a given amount of food patches, the proportion of producers should decrease as the size of the search area increases and the rate of resource discovery, therefore, decreases. The expected proportion of producers should also be higher when the food is dispersed into a large number of poor patches rather than clumped into a few rich patches (Figure 2a). This prediction arises because a scattered distribution implies not only more patches (and then a higher probability of finding a food patch) but also a larger finder's share (i.e., *a*/*F*) that both contribute to increase individuals’ investment in the producer tactic. Finally, if the probability of finding a food patch is independent of the proportion of producers, varying group size does not change the proportion of producers. By contrast, if interactions between the producers either reduce (i.e., when α > 0) or increase (i.e., when α < 0) their efficiency in finding food patches, the model predicts that increasing group size should cause a decline or an increase in the proportion of producers, respectively (Figure 3a).

## 4 A STOCHASTIC SIMULATION MODEL

We used a simulation model to confirm the qualitative predictions from the analytic model regarding the effects of group size (G), patch richness (F), and patch‐discovery rate (*f*) on PS tactic use. As in the analytic model, the probability of discovering a food patch depends on both the number of food patches and the total number of potential patches. To simulate the effect of increasing group size on the time spent searching for food, and hence on the patch‐discovery rate, we also made vary the number of unexploited patches that a producer can assess during a time step.

Specifically, the model simulates a group of G identical individuals searching for food in a foraging area that comprises *N*_SA_ patches, among which only *N_F_*, randomly chosen patches, contain F indivisible food items. A simulation consists of 1,000 time steps during which the ecological conditions remain unchanged to allow the group to converge toward an equilibrium value. Once a food patch is depleted, therefore, it is immediately replaced by a new patch that contains F items and whose position is randomly selected among all empty (with no food) and unoccupied (with no competitor) patches. At each time step, all individuals choose to play producer or scrounger, one after the other. In order to not generate differences among individuals in their tactic use that would result from their initial arrival order on the foraging area (Dubois, Réale, & Giraldeau, 2012), we assume that all individuals play producer in the first time step, thereby searching for their own resources. During a single time step, a producer can assess the quality of *δ* different unexploited patches. We introduced this parameter in the simulation model to make vary the probability of finding a food patch, and, more specifically, to explore the effect of increasing group size on the frequency of producers when the efficiency of producers in finding food patches is larger (i.e., high values of *δ*) or smaller (i.e., small values of *δ*) in larger groups. In the first time step, all individuals can then, in turn, randomly choose a new unoccupied patch until they find a food patch, in which case they get one food item from the patch, or until they have visited *δ* empty patches. At the end of the first time step, all individuals, therefore, can be in one of two possible states: PF (if the individual played producer and got one food item) or PN (if it played producer and got no food).

**Figure 4:**
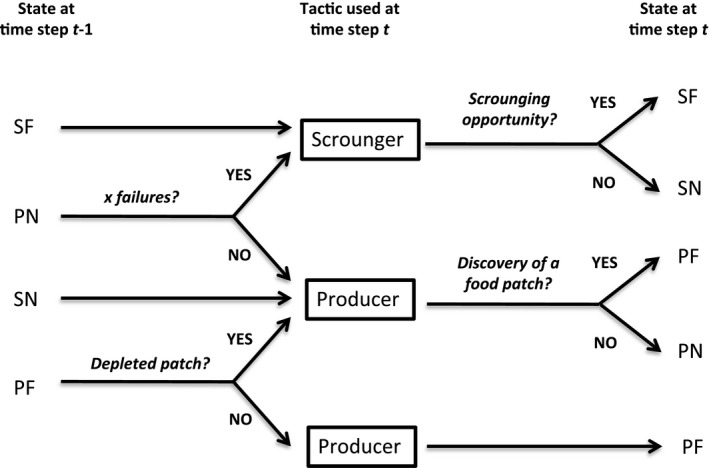
Tactic used by each individual at time *t* (words in black frame) depending on its state (i.e., SF, SN, PF, or PN) at time (*t* − 1)

For each of the subsequent time steps, we used a very simple decision rule and assumed that individuals’ decision is determined solely by their state during the previous time step (Figure 4). More precisely, if an individual obtained a food item in the previous time step, as either a producer (i.e., state PF at time *t* − 1) or a scrounger (i.e., state SF at time *t* − 1), it keeps the same tactic. By contrast, if an individual was unsuccessful as a scrounger (i.e., state SN at time *t* − 1), it switches to producing. Finally, if an individual was unsuccessful at obtaining food as a producer (i.e., state PN at time *t* − 1), it switches to scrounging if it failed to find a food patch during *x* consecutive time steps, or keeps playing producing otherwise. To follow the logic of the analytic model and prevent the scroungers to all exploit the same patch when several scrounging opportunities are available, we assume that an individual playing scrounger can detect without error all joining opportunities (i.e., nonempty patches that are being exploited by at least one competitor), whatever their distance, and will always join the richest patch from which it will get one food item. Thus, even if the patch from which a scrounger obtained food in the previous time step is not depleted yet, it can join another one. By contrast, an individual that was successful at obtaining food as a producer in the previous time step will always stay on the same patch until it is depleted.

For a given set of parameters, each simulation was replicated 100 times to take into account stochastic events, and the frequency of producer tactic use (i.e., number of times individuals used the producer tactic/ total number of times individuals chose one or the other tactic) was averaged across replicates.

## 5 PREDICTIONS

As the analytic model, the stochastic simulation model predicts that the frequency of producers should increase as the number of food patches increases (Figure 1b) but decrease when patch value increases. Consequently, the expected proportion of producers should be higher when the food is dispersed in a large number of poor patches rather than clumped into few rich patches (Figure 2b). Logically, the simulation model also predicts that producer tactic use should increase with the efficiency of producers at finding food and hence with the number of different patches a producer can assess during a single time step. If the number of patches that a producer can assess during a single time step is independent of group size or decreases with the number of competitors, we then predict that increasing group size should decrease the proportion of producers (Figure 3b). By contrast, if increasing group size tends to decrease the time spent in vigilance, thereby allowing individuals to assess the quality of more patches per time unit, the proportion of producers should be higher in larger groups (Figure 3b). Yet, because the number of food patches is the same whatever the size of the group, a slight increase in the number of food patches each producer can sample during a time step does not necessarily increase its probability of finding a food patch. Increasing group size, therefore, may have no immediate impact on the number of simultaneous scrounging opportunities and for that reason may first slightly decrease and then increase the proportion of producers (Figure 3b).

## 6 DISCUSSION

### 6.1 Effects of food distribution and predation risk on PS tactic use

Predictions from the analytic and stochastic simulation models both confirm the empirical findings that individuals invest more in the scrounger tactic when the food is clumped into few rich patches rather than dispersed into many poor patches. This effect is also in agreement with the prediction of earlier models (Barta & Giraldeau, 2000; Giraldeau & Caraco, 2000; Vickery et al., 1991) that the proportion of scrounging should decrease as the finder's share increases (and so as the food becomes more dispersed). The higher frequency of producers in the scattered distribution predicted by our analytic model, however, is not solely the consequence of a larger finder's share when the food patches contain few items but also the consequence of more simultaneous scrounging opportunities and hence fewer scroungers competing for the remainder resource. Both factors, indeed, contribute to increasing the quantity of food that can be obtained by the finder, thereby increasing the benefits to producing. For a given amount of resources available, we would also expect fewer scroungers in environments with low predation pressure. This is because individuals can afford to spend less time looking out for predators but more time searching for food when the risk of predation is lower. As a consequence, producers should assess a larger number of patches per unit of time in environments with low predation risk, leading to an increase in their efficiency at finding food and a decrease in scrounger behavior, as predicted by our simulation model. Again, this effect is in agreement with the prediction of an earlier model by Ranta, Peuhkuri, Hirvonen, and Barnard (1998) but for a different reason. Indeed, Ranta et al.’s model is based on the assumption that producers are more vulnerable to predators than scroungers. In order to minimize the ratio of mortality risk to food intake, it then predicts that individuals should increase their scrounging behavior when the risk of predation increases. Accordingly, Barta, Liker, and Monus (2004) reported that tree sparrows (*Passer montanus*) scrounge more in risky environments (Barta et al., 2004). When vigilance and scrounging are incompatible (Coolen & Giraldeau, 2003), however, this effect cannot be attributable to the antipredatory benefits derived from scrounging, but would rather result from a decrease in the time spent searching for food, as predicted by our analysis.

### 6.2 Effects of group size and interference on PS tactic use

The original PS game predicts that increasing group size should always increase scrounging (Giraldeau & Caraco, 2000; Vickery et al., 1991). Such a prediction arises because increasing group size increases the number of opportunities to join (i.e., the number of sequential discoveries that occur during a given time), if the proportion of producers and scroungers remains fixed, thereby leading to an increase in the scroungers’ expected payoff but to a decrease in the producers’ expected payoff. In accordance with this prediction, some authors found evidence that the proportion of scrounging events was indeed higher in large groups (Aplin & Morand‐Ferron, 2017; Coolen, 2002). By contrast, however, others studies reported either that scrounging decreased rather than increased when group size increased (Haan & Kooreman, 2002; Isaac, Walker, & Williams, 1994) or no effect of group size on the frequency of producers (Liker & Bókony, 2009). Our study can help to explain this discrepancy, as both approaches predict that increasing group size may either exacerbate or mitigate scrounger tactic use. More precisely, when the rate of resource discoveries increases with the number of producers searching for food, as a result for instance of a decrease in the time spent in vigilance, we predict that the proportion of individuals playing scrounger should decrease with increasing group size. In contrast, when the probability of finding a new food patch decreases as forager density increases (Vahl, Meer, Weissing, Dullemen, & Piersma, 2005), increasing group size should cause an increase in the frequency of scrounging. Thus, our model suggests that a decrease in the frequency of producers when group size increases, as demonstrated by a number of experimental studies, would be the consequence (rather than the cause) of a decline in the rate of food patch discovery, because of the interference between individuals searching for food and/or the depletion of the resources.

### 6.3 Importance of variance in the payoffs to producing and scrounging

Our analytic model predicts that foraging groups engaged in a PS game with multiple scrounging opportunities should always converge toward an equilibrium value that corresponds to the best response strategy for a fixed mean number of discoveries. This conclusion holds only if the patches are renewed exactly at the same rate as they are exploited, since otherwise the number of food patches either progressively decreases to zero (if the renewal rate is smaller than the exploitation rate) or continuously increases (if the renewal rate is larger than the exploitation rate), thereby preventing the group to reach an equilibrium value. Under natural conditions, however, animals can often move between foraging groups in order to maximize their success, which would contribute in stabilizing the amount of available resource within foraging areas. Models of multi‐level selection could tackle this question and further address the impact of scrounging on resource sustainability. Nevertheless, when the ecological conditions remain unchanged, thereby allowing the group to reach an equilibrium value, the number of opportunities actually available is likely to change over time because of stochastic effects, which might prevent individuals to accurately assess their number, and hence cause fluctuations in PS tactic use. Such fluctuations around the expected equilibrium value should be more pronounced when the variance of the mean number of food discoveries is large, that is, for instance, when the rate of food discoveries is intermediate. Contrary to the view that foraging groups engaged in a PS game should always converge to a stable equilibrium (Afshar & Giraldeau, 2014; Giraldeau & Caraco, 2000; Valone et al., 2017), our model thus suggests that it might not be the case when the variability in the payoffs to producing and scrounging is large. Variability in the payoffs may be caused by changes over time in the number of simultaneous discovered patches but also, for instance, in the size of the food patches or in the distance between the scroungers and the discovered patches. This idea is supported by experimental evidence that the magnitude of fluctuations around the equilibrium frequency of scrounging may vary substantially across conditions. For instance, results from Morand‐Ferron and Giraldeau’s (2010) experiments clearly indicate that scrounging rates are more variable when the food is clumped into a small number of rich patches compared with when it is dispersed, probably because the quantity of food an individual can obtain from a patch, which may change depending on the number of scroungers and/or the time before they join the patch, is more variable when food patches are larger. These findings suggest that the existence of payoff variability may not only lead to suboptimal tactic use but also would contribute in individual differences in tactic use.

In conclusion, the generalization of the PS game to multiple simultaneous scrounging opportunities not only demonstrates that certain ecological factors that were considered unimportant are key predictors of free riding but also provides in several cases an alternative explanation for the changes in producer and scrounger tactic use that may account for contradictory empirical findings. Furthermore, our analytic model suggests that ecological conditions that yield greater variance in the payoffs to producing and scrounging could cause fluctuations in tactic use and prevent groups to converge toward a stable equilibrium. Because free riding contributes to reduce the speed of resource exploitation, it may be highly relevant to predict accurately its frequency when individuals exploit renewable resources, hence the importance of considering the effect of simultaneous scrounging opportunities.

## CONFLICT OF INTERESTS

The authors declare they have no competing interests.

## AUTHORS’ CONTRIBUTIONS

FD and ER‐D designed the study and wrote the paper, and FD developed the models.

## Supporting information

Supplementary Material

## Data Availability

The code of the simulation model implemented in C++ is available in Dryad (https://doi.org/10.5061/dryad.prr4xgxhq).
