# Supplementary material for: Consequences of multiple simultaneous opportunities to exploit others’ efforts on free riding
Source: Ecol Evol. 2020 Apr 16;10(10):4343–51. doi: 10.1002/ece3.6201 (PMC7246214; doi:10.1002/ece3.6201)
Supplement: Supplementary file 1 — Supplementary Material [file ECE3-10-4343-s001.docx]

**Supplementary material S1: Code of the simulation model implemented in C++**

#include<stdio.h>

#include<stdlib.h>

#include<math.h>

#include<time.h>

FILE*fichier_simul;

void main (void){

const int F=20;

const int nf=20;

const int N=400;

const G=10;

const int tentative_max=5;

const max_echec=5;

const int nb_simul=100;

const int T=1000;

int t,i,j,s,VA,VP;

int tentative;

int value[1000];

int statut[1000];

int nb_foragers[1000];

int gain_attendu[1000];

int gain_max;

int position_attendue;

int gain_produce, gain_scrounge, nb_produce, nb_scrounge,cumul_opportunite;

int strategy[100];

int etat[100];

int position[100];

int echec[100];

int opportunite;

srand(time(NULL));

fichier_simul=fopen("Resultats.txt","w");

for(s=0;s<nb_simul;s++){

cumul_opportunite=0;

for(i=0;i<N;i++){

value[i]=0;

nb_foragers[i]=0;

gain_attendu[i]=0;

}

for (i=0;i<nf;i++){

do{

VA=(rand()%N);

}

while(value[VA]!=0);

value[VA]=F;

}

gain_produce=0;

gain_scrounge=0;

nb_produce=0;

nb_scrounge=0;

/* TIME STEP 0*/

for(i=0;i<G;i++){

strategy[i]=1;

etat[i]=0;

echec[i]=0;

do{

VA=(rand()%N);

}

while(nb_foragers[VA]!=0);

position[i]=VA;

nb_foragers[position[i]]+=1;

if(value[position[i]]>0){

etat[i]=1;

gain_produce+=1;

value[position[i]]-=1;

if(value[position[i]]==0){

do{

VP=(rand()%N);

}

while((value[VP]!=0)||(nb_foragers[VP]!=0));

value[VP]=F;

}

}

else if (value[position[i]]==0){

echec[i]+=1;

if (echec[i]>=max_echec){

strategy[i]=2;

echec[i]=0;

}

}

}

/* TIME STEP 1 TO T*/

for(t=0;t<T;t++){

for (i=0;i<G;i++){

if (strategy[i]==1){

nb_produce+=1;

tentative=0;

if((etat[i]==1)&&(value[position[i]]>0)){

gain_produce+=1;

value[position[i]]-=1;

if(value[position[i]]==0){

do{

VP=(rand()%N);

}

while((nb_foragers[VP]!=0)||(value[VP]!=0));

value[VP]=F;

}

}

else{

nb_foragers[position[i]]-=1;

etat[i]=0;

for(j=0;j<N;j++) statut[j]=0;

do{

VA=(rand()%N);

if((nb_foragers[VA]==0)&&(statut[VA]==0)){

statut[VA]=1;

tentative+=1;

}

}

while((tentative<=tentative_max)&&(value[VA]==0));

position[i]=VA;

nb_foragers[position[i]]+=1;

if(value[position[i]]>0){

etat[i]=1;

echec[i]=0;

gain_produce+=1;

value[position[i]]-=1;

if(value[position[i]]==0){

do{

VP=(rand()%N);

}

while((value[VP]!=0)||(nb_foragers[VP]!=0));

value[VP]=F;

}

}

else if (value[position[i]]==0){

etat[i]=0;

echec[i]+=1;

if (echec[i]>=max_echec){

strategy[i]=2;

echec[i]=0;

}

}

}

}

else if (strategy[i]==2){

nb_scrounge+=1;

opportunite=0;

gain_max=-10;

position_attendue=position[i];

for(j=0;j<N;j++){

if((nb_foragers[j]>0)&&(value[j]>0)){

opportunite+=1;

cumul_opportunite+=1;

if(value[j]>gain_max){

gain_max=value[j];

position_attendue=j;

}

}

}

if(opportunite>0){

nb_foragers[position[i]]-=1;

position[i]=position_attendue;

nb_foragers[position[i]]+=1;

etat[i]=1;

gain_scrounge+=1;

value[position[i]]-=1;

if(value[position[i]]==0){

do{

VA=(rand()%N);

}

while((nb_foragers[VA]!=0)||(value[VA]!=0));

value[VA]=F;

}

}

else if(opportunite==0){

strategy[i]=1;

etat[i]=0;

}

}

}

}

fprintf(fichier_simul,"\n%d\t %d\t %d\t %d\t %d\t",nb_produce,nb_scrounge, cumul_opportunite, gain_produce, gain_scrounge);

}

fclose(fichier_simul);

}
